# Management of complicated giant prostate hyperplasia

**DOI:** 10.4314/gmj.v59i2.7

**Published:** 2025-06

**Authors:** Mathew Y Kyei, Ernest K Agyapong-Tandoh, Esinam A Amedewonu, James E Mensah, Josephine M Kyei, Seth Oti-Mensah

**Affiliations:** 1 Department of Surgery and Urology, University of Ghana Medical School, Accra, Ghana; 2 Department of Surgery, Korle Bu Teaching Hospital, Accra, Ghana; 3 Department of Anaesthesia, Korle Bu Teaching Hospital, Accra, Ghana; 4 School of Nursing and Midwifery, University of Ghana, Legon, Accra, Ghana; 5 Trust Specialist Hospital, Accra, Ghana

**Keywords:** giant prostate hyperplasia, haemorrhage, surgery, open prostatectomy, case report

## Abstract

Giant prostate hyperplasia is defined as prostate hyperplasia with a weight greater than 500 grams. This condition is rare. We present the management of a case of giant prostate hyperplasia (541 grams on CT scan) with a surgical enucleated volume of 800 grams in a patient who presented with haematuria. An abdominal-pelvic CT scan was required to differentiate it from a suspected bladder tumour. This case was successfully managed with a favourable outcome following open transvesical prostatectomy, ensuring minimal blood loss. This case report and review provide an update on the management of giant prostate hyperplasia, with emphasis on the prevention and management of haemorrhage.

**Funding:**

None declared

## Introduction

Giant prostatic hyperplasia, defined as a prostate adenoma weighing over 500g,^1^ is rare, with surgical management typically required when complicated by hematuria.^2^ We present the management of a case of giant prostatic hyperplasia, measuring 541g on CT scan and with a surgical enucleated volume of 800g, focusing on perioperative haemorrhage prevention and management. The patient provided written consent for publication.

## Case Report

A 65-year-old of African descent presented with a one-year history of obstructive lower urinary tract symptoms that worsened despite phytotherapy. He also experienced recurrent haematuria with blood clots. The patient, an office worker with no smoking history, had a father who experienced lower urinary tract symptoms due to BPH around age 60 but did not require surgery. The examination revealed that he was afebrile and not pale. Abdominal examination showed a firm, non-tender suprapubic mass, while digital rectal examination indicated a markedly enlarged prostate with a non-palpable median sulcus.

Due to hematuria and the suprapubic mass, an abdominal-pelvic CT scan was requested alongside other investigations. On the morning of the scan, the patient experienced suprapubic pain. Suspecting clot retention, an attempt at urethral catheterization was made, but no urine was obtained, and the catheter was left in place.

**Figure 1:**
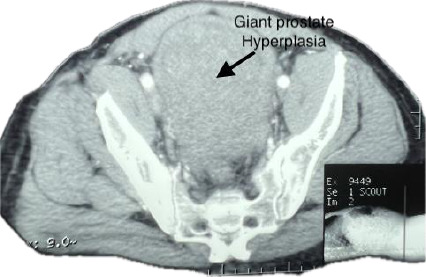
Pelvic CT scan showing giant hyperplasia of the prostate, estimated weight 541g (10.2 × 8.3 × 12.3 cm)

Investigations showed a haemoglobin level of 11.0 g/dl. Blood, urea, and electrolyte levels revealed sodium 139.3 mmol/L, potassium 4.66 mmol/L, urea 2.4 mmol/L, and creatinine 103.78 µmol/L, with an eGFR of 75 mL/min/1.73 m^2^. Total prostate-specific antigen (PSA) was 36.96 ng/ml. The abdominal-pelvic CT scan showed a grossly enlarged prostate weighing 541g, with associated blood clots in the urinary bladder and no hydronephrosis (Figure 1). The Foley catheter tip was located in the prostatic urethra.

Given the suspicion of clot retention, the patient was counselled and consented to a transvesical open prostatectomy to inspect the urinary bladder for other pathologies. The surgical technique used was a Freyer's suprapubic transvesical prostatectomy^2^ under spinal anaesthesia, with 1g of intravenous tranexamic acid administered.

**Figure 2:**
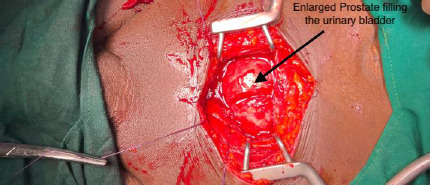
Prostate hyperplasia filling the urinary bladder

The findings revealed a significantly enlarged prostate, with the middle lobe extending into the bladder (Figure 2), accompanied by bladder wall hypertrophy.

**Figure 3:**
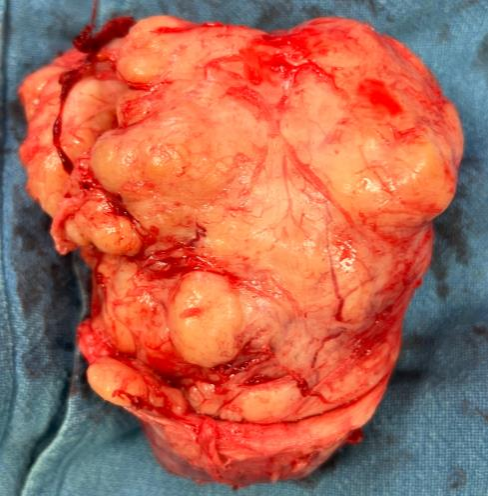
Enucleated giant prostate hyperplasia

The prostatic adenoma was enucleated as a single piece, weighing 800 grams (Figure 3). Haemostasis was achieved using 0 Vicryl sutures at the 5 o'clock and 7 o'clock positions at the bladder neck, approximating the bladder wall to the prostate capsule continuously.

Packing of the adenoma fossa was not performed. The estimated blood loss was 350mls, and the patient was transfused with one unit of blood. A size 24 three-way Foley catheter was inserted after achieving haemostasis, and traction was applied.

The cystostomy (bladder incision) was closed using 1 Vicryl suture, ensuring mucosa-to-mucosa approximation to enhance haemostasis by minimising blood loss from the incised detrusor muscles. A retropubic drain was placed, and the wound was closed with 1 Vicryl for the fascia and interrupted nylon for the skin. The patient received bladder irrigation to prevent clot retention and was prescribed oral oxybutynin (5mg three times a day for seven days) to manage bladder spasms associated with cystostomy in a hypertrophied bladder wall.

The urethral catheter was removed on postoperative day 8, and the patient was discharged, voiding well without any gross haematuria. The patient expressed satisfaction with the outcome of his management. Histology of the enucleated adenoma indicated nodular fibromyoglandular hyperplasia, and a repeat PSA seventeen days post-surgery was 3.72 ng/ml.

## Discussion

Prostates larger than 100g are found in approximately 4% of men over 70 years old.^3^ Giant prostatic hyperplasia exceeding 500g is rare, with few reported cases. The cause is attributed to the exaggerated expression of growth factors and mutations in proto-oncogenes and p53 suppressor genes.^2^,^5^ Clinical presentations may include lower urinary tract symptoms, acute urinary retention, haematuria, obstructive nephropathy,^2^,^6^,^7^ and bilateral pedal oedema.^8^

This case presented with lower urinary tract symptoms and hematuria, with the giant adenoma observable as a suprapubic mass on examination. Its smooth and firm surface distinguishes it from bladder tumours, which typically have irregular surfaces. Digital rectal examination findings revealed a firm, enlarged prostate supporting its benign features. Elevated PSA levels are common in large prostates, as seen in our case, which recorded 36.96 ng/ml. In elective cases, a prostate biopsy is recommended to exclude cancer;^6^,^8^ however, in emergencies, histological assessment of the enucleated adenoma is sufficient.

Follow-up PSA tests are essential to confirm normalisation post-surgery.^7^ Ultrasound and CT scans help delineate the prostate.^8^ In cases with haematuria, abdominalpelvic CT scans and CT-IVU can help exclude malignant urinary tract lesions. However, cystoscopy may be limited by the size of the adenoma.

In this case, the rigid cystoscope could not advance due to length limitations and extreme angulation, causing laceration of the prostate middle lobe and subsequent bleeding.

Laparoscopic and robotic-assisted techniques have shown limited success in larger prostates,^9^,^10^, while open retropubic and transvesical approaches have been effective.^2^,^7^,^8^,^11^ The case presented had an open transvesical prostatectomy, which had an additional advantage of allowing inspection of the urinary bladder for any other pathology, as the patient presented with haematuria. In cases of giant prostatic hyperplasia with recurrent haemorrhage, prostate arterial embolisation may reduce haematuria, though lower urinary tract symptoms may worsen.^6^

Haemorrhage leading to hemodynamic instability is a significant concern during surgery for giant prostate adenoma, with a rapid procedure noted to minimise blood loss.^2^ The intraoperative administration of antifibrinolytic agents such as intravenous tranexamic acid has been shown to reduce blood loss in prostate surgery.^12^ Using absorbable sutures at the 5 o'clock and 7 o'clock positions for haemostasis and approximating the bladder mucosa to the prostate capsule can expedite haemostasis.^13^,^14^ Bladder irrigation and larger three-way Foley catheters (24F) with large retention balloon reservoirs enhance haemostasis when put under traction.^15^ The placement of a suprapubic catheter, in addition to a urethral catheter, enhances bladder irrigation that prevents clot retention.

In cases of persistent bleeding where intraoperative control fails, purse-string closure of the bladder neck, as described by Malament,^16^ can be employed. If bleeding remains excessive, locating and ligating the internal iliac arteries may be necessary.^2^,^17^ Contemporary surgical management of giant prostatic hyperplasia has improved outcomes, with focus on hemostasis and timely blood transfusions as key factors in reducing perioperative mortality.^18^,^19^,^20^

## Conclusion

Open surgery is the preferred approach for managing complicated giant prostatic hyperplasia. Employing effective techniques for rapid surgery and maintaining hemostasis ensures satisfactory outcomes.
